# Acute Dissection of the Ascending Aorta as a Rare Complication of Aortocoronary Bypasses Surgery: A Case Report

**Published:** 2019-10

**Authors:** Ferhat Borulu, Bilgehan Erkut

**Affiliations:** *Department of Cardiovascular Surgery, Medical Faculty, Atatürk University, Erzurum, Turkey. *

**Keywords:** *Coronary artery bypass*, *Aneurysm, dissecting*, *Aorta*

## Abstract

Many complications may ensue coronary bypass surgery. Among these complications, early or late dissection of the ascending aorta is a very rare condition. Successful surgery without life-threatening results can save lives. In this case report, we present a case of aortic dissection due to cross-clamp injury in a patient who underwent coronary artery bypass surgery.

A 60-year-old female patient underwent double-vessel aortocoronary bypass surgery due to coronary artery disease. After distal bypasses were performed and the cross-clamp was removed, dissection of the aorta occurred. Consequently, axillary cannulation and ascending aortic replacement with a prosthetic graft were performed via the open technique. The saphenous vein graft was sutured to the aortic prosthetic graft, and cardiopulmonary bypass was terminated after hemodynamic stability was achieved. The patient was discharged on the 10th postoperative day, and she has been followed up for 2 years after surgery without any problems.

## Introduction

Aortocoronary bypass surgery has been an accepted treatment option for many years in all patients with coronary artery disease. Myocardial infarction, pericardial tamponade, pericarditis, and postoperative infection are some of the more frequently encountered complications. However, dissection of the ascending aorta during coronary artery bypass grafting (CABG) is an uncommon but potentially fatal complication of cardiac surgical procedures.^1^^, ^^2^

May conditions may cause aortic injury and associated aortic dissection; these include severe atherosclerotic changes in the aortic intima, aorta to thin or dilated ascending aorta, cross-clamp injury, cystic medial necrosis, and collagen vascular disease.^2^^-^^4^ Preoperative aortic evaluation and appropriate surgical techniques in risky patients may prevent aortic dissections that may be associated with cross-clamp injury. We report a case of the acute dissection of the ascending aorta after cross-clamping removal during CABG and its subsequent successful repair.

## Case Report

After coronary angiography, a 60-year-old female patient was taken to the cardiovascular surgery department for CABG. The patient had no cardiac pathology other than coronary artery disease and hypertension. Telecardiographic imaging was normal. Echocardiography demonstrated normal cardiac cavities, normal valve functions, an ejection fraction of 55%, and an ascending aorta diameter (3.1 cm) within the normal limits. Angiography showed obstructions in the left anterior descending and circumflex arteries, which required surgical intervention. 

**Figure 1:**
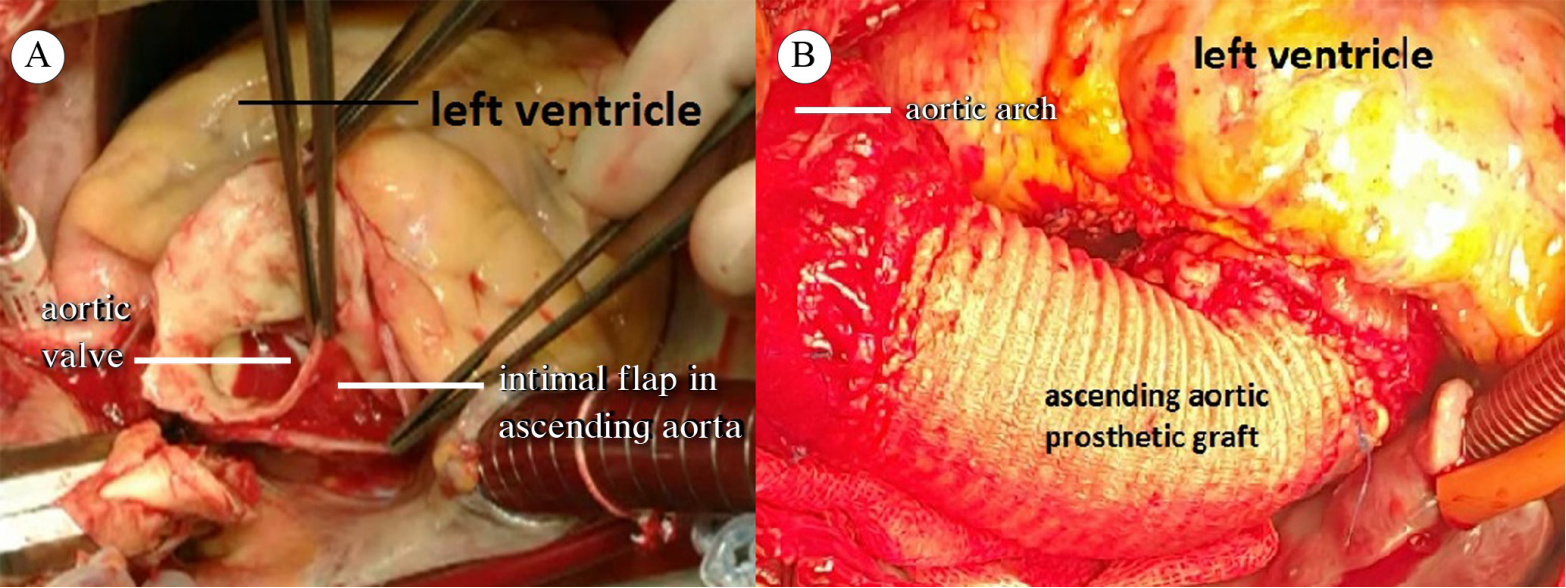
A) Perioperative image shows the aortic dissection and the intimal flap in the ascending aorta, thought to be related to cross-clamp injury. B) This image shows a completed surgical image of ascending aorta replacement with prosthetic graft material due to the aortic dissection.

**Figure 2:**
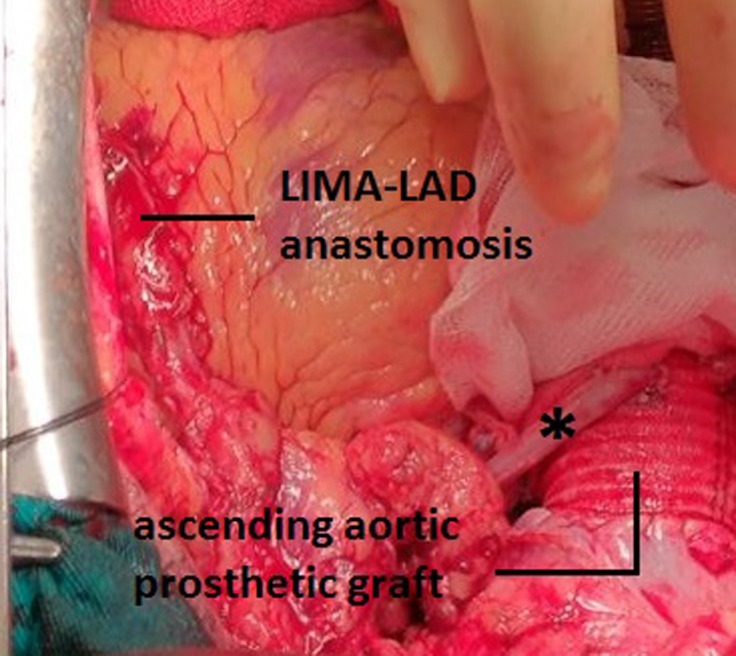
This image shows the anastomosis of the saphenous vein sutured to the circumflex artery to the ascending aortic prosthetic graft (black asterisk), as well as the LIMA-LAD anastomosis.

After preoperative preparations, the patient underwent surgery. Cardiopulmonary bypass (CPB) was established through aortic-caval cannulation after classical sternotomy. The ascending aorta was soft, flexible, and of normal size. After lowering the temperature to 32 ^°^C, a cross-clamp was placed on the ascending aorta. The left anterior descending and circumflex arteries were bypassed with the internal mammary artery and the saphenous vein, respectively. The patient’s systolic blood pressure was maintained at 40–50 mmHg during the coronary bypass distal anastomoses. After the completion of the distal anastomoses, the aortic cross-clamp was removed. After the removal of cross-clamping, damage was detected in the ascending aorta. The ascending aorta expanded and its color changed. Cross-clamp-related injury and aortic dissection were considered. Urgently, an arterial cannula was inserted into the right axillary artery without removing the arterial cannula in the aorta. Then, the patient was separated from CPB. The arterial line from the heart-lung machine was separated from the aortic cannula and inserted rapidly into the axillary artery cannula. Thereafter, CPB was resumed. The arterial cannula on the ascending aorta was removed. The innominate artery was clamped, so that cerebral perfusion was continued. The patient was cooled down to 28 ^°^C. The ascending aorta was dissected via the open technique. There was an injury of approximately 4 mm in the intimal layer in the region where the cross-clamp was placed in the ascending aorta, and the proximal and distal intimae were separated from the media layer (Figure 1A). An intimal irregularity at the site of the intimal dissection, together with an atherosclerotic plaque with calcification, was observed. Primary repair or patchplasty was not deemed appropriate because the dissection flap had advanced into proximal and distal directions due to the intimal injury. The dissecting region of the ascending aorta and the calcified aortic tissues in the proximal and distal parts of this region were excised. Ascending aortic replacement was performed using a 28-Dacron prosthetic graft (Figures 1B). The anastomoses were strengthened with fibrin glue. The innominate artery clamp was removed, and total circulation was resumed. Proximal anastomosis was made to the ascending prosthetic graft with 4/0 PROLENE continuous sutures using side-clamping (Figure 2). The patient was re-warmed. Weaning from CPB was uneventful and required a minimal dopamine infusion. She was stable without any problems in the intensive care unit. Her postoperative course was remarkably uneventful. Histologic examination of the resected aorta revealed chronic inflammation, atherosclerosis, and thrombus. The patient was discharged on the 10th postoperative day. She underwent surgery on March 10, 2017, and has been followed up for 2 years, with her last control date being March 19, 2019.

## Discussion

Ascending aortic dissection during and after CABG is a rare but potentially fatal complication in surgical patients. Unless the dissection is immediately diagnosed and treated, the patient faces a life-threatening situation.

Aortic dissection may occur spontaneously due to pathological conditions such as hypertension, trauma, and medial necrosis; nonetheless, dissection may occur iatrogenically during and after cardiac surgery. The medical literature contains many cases of aortic dissection that occurred during and after cardiac surgery.^1^^-^^5^ The aim in such patients, whether intraoperative or postoperative, is to make the diagnosis as soon as possible and to reduce morbidity and mortality with successful surgery.

Intra- and post-operative aortic dissection can usually occur due to aortic cannulation, the use of aortic cardioplegia needles, cross-sectional or side-clamp application areas, or anastomoses to the ascending aorta (CABG proximal anastomoses).^6^^, ^^7^ Such invasive procedures applied directly on the ascending aortic wall can cause damage, tearing, and finally dissection.^1^^, ^^8^ Aortic dissection cases during CABG have been encountered by many surgeons and have been reported over the years.^2^^, ^^8^^, ^^9^ Uncontrolled hypertension, cystic media-necrosis, and/or atherosclerosis have been reported to be important risk factors leading to aortic dissection during open-heart surgery.^1^^, ^^3^ Furthermore, intimal injury can occur due to damage to the aortic tissue. In our patient, dissection occurred in the cross-clamp region. We believe that the cross-clamp caused damage and led to dissection in an atherosclerotic and weak region of the aortic intima. As is explained in the Laplace law, increased intraluminal tension and blood pressure may exert an effect on the aorta. Therefore, both atherosclerosis and hypertension are known to lead to the development of aortic dissection.^8^^, ^^9^ What should not be ignored in this regard is that a previously known disease of the aortic wall, sutures, cannulation, or cross-clamp application may become the triggering mechanism. If the presence of atherosclerotic and calcified plaques is detected in the ascending aorta by hand examination, it can help the surgeon to choose more carefully the sites of the aortic cannula, saphenous vein anastomosis, and cross-clamping. Our patient had a history of hypertension; still, we could not find a significant plaque formation in the aorta by manual examination at the time of cannulation. Accordingly, the prevention of early postoperative dissection in CABG requires that surgery be completed considering several factors in relation to the aorta: avoiding aortic clamping as beating heart, maintaining a low systemic blood pressure by pharmacologic means during proximal anastomoses (systolic blood pressure <80 mmHg during proximal anastomoses), and avoiding the use of partial occluding clamps as much as possible. In addition, the ascending aorta can be replaced with prosthetic grafts in ascending aorta cases with dilated, calcified, or diffuse atherosclerosis.

Since intraoperative aortic dissection is a high-risk and mortal condition, patients should be kept under observation until the end of the operation in terms of pathological appearance in the aortic wall. As soon as the diagnosis is made, urgent repair is required to prevent dissection enlargement and complications. The shape of the repair depends on the location and width of the intimal tear. Primary repair is the shortest possible treatment option in patients with localized dissection. Patchplasty or graft interposition should be performed rapidly in patients with extensive damage. When dissection occurs, rapid diagnosis and appropriate surgical treatment are required to improve the patient’s outcome.

## Conclusion

A rare but devastating complication of cardiac operations is perioperative iatrogenic dissection originating in the ascending aorta, which occurs with an incidence of 0.16%–0.6%. Clinical awareness of aortic dissection after CABG in this group of patients should make early diagnosis with successful surgical correction feasible.

## Notes:

***This paper should be cited as: ***Borulu F, Erkut B. Acute Dissection of the Ascending Aorta as a Rare Complication of Aortocoronary Bypasses Surgery: A Case Report. J Teh Univ Heart Ctr 2019;14(4):191-194.
